# The power of positive thinking: Pathological worry is reduced by thought replacement in Generalized Anxiety Disorder

**DOI:** 10.1016/j.brat.2015.12.017

**Published:** 2016-03

**Authors:** Claire Eagleson, Sarra Hayes, Andrew Mathews, Gemma Perman, Colette R. Hirsch

**Affiliations:** a King's College London, Institute of Psychology, Psychiatry and Neuroscience, London, UK; b Curtain University, Perth, Australia; c University of California, Davis, USA; d Berkshire Healthcare NHS Foundation Trust, UK

**Keywords:** Generalized anxiety disorder, Worry, Imagery, Verbal processing, Positive thoughts

## Abstract

Worry in Generalized Anxiety Disorder (GAD), takes a predominantly verbal form, as if talking to oneself about possible negative outcomes. The current study examined alternative approaches to reducing worry by allocating volunteers with GAD to conditions in which they either practiced replacing the usual form of worry with images of possible positive outcomes, or with the same positive outcomes represented verbally. A comparison control condition involved generating positive images not related to worries. Participants received training in the designated method and then practiced it for one week, before attending for reassessment, and completing follow-up questionnaires four weeks later. All groups benefited from training, with decreases in anxiety and worry, and no significant differences between groups. The replacement of worry with different forms of positive ideation, even when unrelated to the content of worry itself, seems to have similar beneficial effects, suggesting that any form of positive ideation can be used to effectively counter worry.

## Highlights

• People with GAD practiced replacing worry with alternatives for one week.
• Alternatives were positive outcomes rehearsed as images or verbal thoughts.
• A control group rehearsed positive images unrelated to their worries.
• One month later all groups reported significantly reduced anxiety and worry.
• Unexpectedly, even unrelated positive ideation can effectively counter worry.

## 1 Introduction

Excessive worry is a common symptom in anxiety disorders and is the central feature of Generalized Anxiety Disorder (GAD). In Hirsch and Mathews' (2012) model of pathological worry three processes combine to maintain uncontrollable worry: emotional processing biases, impaired attentional control and the tendency to represent possible negative outcomes in over-general verbal form. The aim of the study reported here was to investigate the effects of different methods designed to modify this last process in pathological worriers with GAD.

Worry is predominantly verbal, as if talking to oneself about possible negative outcomes, whereas imagery is relatively infrequent, and tends to be brief (Freeston et al., 1996, Hirsch et al., 2012). In contrast, when instructed to relax, non-worriers report primarily images whereas those with GAD report similar amounts of verbal thought and imagery (Borkovec & Inz, 1990). The latter authors suggested that verbal worry may be a strategy to avoid more distressing emotional representations, such as images (Borkovec, Alcaine, & Behar, 2004). In partial support of this idea, Butler, Wells, and Dewick (1995) found that instructions to worry (verbally) about a distressing film led to less anxiety immediately afterwards than did instructions to think about it in images. However, verbal worry led to *more* intrusive images in the days following than did thinking in images. Thus, even if verbal worry leads to temporary reductions in anxiety, it can maintain negative thought intrusions in the longer term.

Similarly, high worriers given instructions to worry verbally reported increased negative thought intrusions from pre- to post-worry, but those instructed to worry in images actually showed a decrease (Stokes & Hirsch, 2010). This suggests that verbal thinking style plays a causal role in maintaining intrusions, perhaps serving to trigger subsequent worry episodes. The question of *why* verbal-based worry elevates intrusive thoughts remains unanswered. One possibility is that verbal thoughts in worry tend to be relatively abstract and over-general, raising many vague possibilities but reducing the possibility of resolving them because they are not clearly defined (Stöber, Tepperwien, & Staak, 2000), which may instead maintain perceived threat (Philippot, Baeyens, & Douilliez, 2006).

Alternatively, increased intrusive thoughts may arise from the detrimental effects of verbal worry on attention and attentional control (Stefanopoulou, Hirsch, Hayes, Adlam, & Coker, 2014). Leigh and Hirsch (2011) demonstrated that verbal worry in high worriers impairs attentional control (compared to non-worriers), but this group difference disappears after worrying using images. Furthermore, Williams, Mathews, and Hirsch (2014) demonstrated that verbal worry increased attentional bias towards threat, but worrying in imagery did not. This evidence suggests that verbal-based worry can maintain intrusive thoughts about threats, in contrast to imagery-based worry.

So far we have only considered thinking about *negative* (worry-related) rather than positive topics. Encouraging imagery of alternative *positive* outcomes might be particularly helpful, by competing in affective valence with the usual negative content of worry. Indeed, Hirsch, Perman, Hayes, Eagleson, and Mathews (2015) found that practice in thinking about worry topics in more positive ways (whether verbally or in images) reduced subsequent intrusions compared with worry in verbal form, although this reduction was not significantly greater than that seen following similar practice using imagery of negative outcomes. However, only practice in thinking about alternative *positive* outcomes (whether as images or in verbal form) also reduced the rated cost of worry outcomes and increased perceived ability to cope with them. Thus it seems likely that practice with positive representations has benefits beyond those produced by worry-related imagery alone.

Alternatively, it could be that verbal worry is best countered by generating opposing positive thoughts in the same (verbal) modality, because this would more directly compete with the negative outcomes rehearsed in worry. It may be, for example, that worry-related intrusions (in verbal form) are more likely to prime alternative positive verbal outcomes that were rehearsed earlier, in comparison to positive images, which would require an additional shift from a semantic to a perceptual modality.

This is the first study to investigate whether extended practice with positive alternatives to worry, either in verbal or imagined form, has lasting effects on anxiety and worry in GAD. Volunteers with GAD were allocated either to practice in replacing the usual form of worry with *images* of positive outcomes, or in which positive outcomes were represented in *verbal* form. Both conditions tested the hypothesis that rehearsing positive outcomes for worry-related concerns should counter negative expectations and reduce worry. However, reductions in worry could conceivably result from replacing negative content with *any* form of positive ideation, whether or not designed to challenge the negative meanings rehearsed in worry. If so, similar effects would follow practice in replacing worry with positive ideation *unconnected* with worry content. Accordingly, in a third (control) condition participants were instructed to practice positive images unrelated to their worry.

## 2 Method

### 2.1 Overview of design

Volunteers with GAD were randomly allocated to one of three conditions: (i) practice in generating mental images of positive outcomes to worry topics (positive imagery of worry, PIW); (ii) practice in generating verbal descriptions of positive worry-related outcomes (positive verbal representations of worry, PVW); or (iii) practice in generating positive images unrelated to any current concerns (positive imagery of non-worry, PIN). All participants completed an initial face-to-face training session, followed by a week of practice in their assigned condition exercise at home, before returning for a post-training assessment session and then completing follow-up questionnaires one month later.

### 2.2 Participants

Participants were volunteers aged 18 to 65, recruited from the community via advertisements, who met criteria for GAD on the Structured Clinical Interview for DSM-IV Axis I (SCID-I; First, Gibbon, Spitzer, & Williams, 1996), and who had English as their first language. Exclusion criteria included a history of Bipolar Disorder or psychosis, and current psychological therapy (past therapy was acceptable). If participants were taking medication for anxiety or depression, they had to be on a stable dose for at least one month prior to taking part. One hundred and fifty participants were initially recruited and attended the first assessment session. Of those, 21 were excluded as not meeting criteria for GAD and one who had just begun Cognitive Behavior Therapy.

Of the 128 participants who met criteria, 26 were excluded: two for not following instructions during initial practice; four failed to attend for reassessment; two did not return follow-up questionnaires; six completed less than 50% of their assigned practice; and 12 reported being unable to think as instructed for most of the time during a check in session 2. A chi-square goodness-of-fit analysis revealed no significant differences in the number excluded across groups, *χ*^2^ (2, *n* = 128) = 1.56, *ns*. Analyses were conducted on data from the remaining participants (32 PIW, 35 PVW and 35 PIN).

Participant characteristics are shown in Table 1. There were no group differences in gender, *χ*^2^ (2, *n* = 102) = 1.43, *ns;* age, *F* (2, 99) = .48, *ns*; education, *F* (2, 99) = .79, *ns*; Penn State Worry Questionnaire scores (PSWQ; Meyer, Miller, Metzger, & Borkovec, 1990), *F* (2, 99) = 1.71, *ns;* State Trait Anxiety Inventory-Trait (STAI-T; Spielberger, Gorsuch, Lushene, Vagg, & Jacobs, 1983), *F* (2, 99) = .49, *ns*; Life Orientation Test-Revised (LOT-R; Scheier, Carver, & Bridges, 1994), *F* (2, 99) = .50, *ns*; Beck Depression Inventory-II (BDI-II; Beck, Steer, & Brown, 1996), *F* (2, 99) = 2.11, *ns*, or number of negative intrusions during the initial breathing focus task (see below), *F* (2, 99) = .22, *ns*.

### 2.3 Breathing focus task

This task was adapted from the Worry Task (Hayes, Hirsch, & Mathews, 2010; Hirsch, Mathews, Lequertier, Perman, & Hayes, 2013). Participants were asked to focus on their breathing for five minutes, and when a tone sounded (on 12 random occasions), to indicate whether they were thinking about their breathing, or something else. If the latter, they classified their thought as positive, negative, or neutral, and gave a brief summary of its content (e.g. “positive: going out tonight”). At the end of the task, participants recorded a fuller description of each thought, allowing an independent assessor, blind to thought origin, to categorize each as positive, negative, or neutral. Negative thoughts were further classified as low, medium or high in negativity. Another assessor classified 25% of participants' reports (*n* = 30; ten per group selected at random); inter-rater reliability for valence classification using Cohen's kappa statistic (κ) was .75, and for negativity .71.

### 2.4 Instructions for each intervention condition

#### 2.4.1 Positive imagery of worry (PIW)

Participants identified three current worry topics and rated how distressing each was; how often they had worried about it over the past week, and how controllable it was, using 0–10 scales. The difference between verbal thoughts and images was then described, and participants informed they would be practicing thinking in mental imagery rather than verbally. They were then asked to produce a vivid image regarding friendship and hold it for 30 s, re-focusing on imagery if verbal thoughts occurred, and then rated how well they had thought in their designated thinking style (imagery), from 0 (not at all) to 10 (completely), and the percentage of thoughts that were positive, negative or neutral. Participants then read a worry description (concerns about paying a bill), and were asked to think of how the scenario could have a positive outcome. For two more descriptions they were instructed to generate vivid images of the situation turning out positively (for one and two minutes respectively), with ratings as before, used by the experimenter to encourage appropriate imagery.

Finally, participants generated lists of potential positive outcomes for each of their own three worry topics, followed by imagining each for two minutes. Designated thinking style and valence ratings were collected as a manipulation check.

#### 2.4.2 Positive verbal worry (PVW)

Instructions were as for PIW, but instead of imagery participants were instructed to think (and then write down) verbal descriptions, and rate their use of verbal mentation (0–10). If participants reported any images, they were instructed to refocus their minds on verbal thoughts.

#### 2.4.3 Positive imagery non-worry (PIN)

This was the same as the positive imagery of worry condition, except that instead of an image of positive worry-related outcomes, participants were instructed to imagine something positive that was completely *unrelated* to the worry scenario they had read, or to any of their own worry topics.

### 2.5 Procedure

Participants were sent the PSWQ and STAI-T to complete and bring to the first session, which they began by completing the BDI-II and the LOT-R, and after a 45 s practice, the breathing focus task, followed by the GAD module of the SCID-I. Participants then completed the designated mentation style training and were instructed to use this style anytime they noticed themselves worrying during the following week. They were also asked to repeat the imagery/verbal practice with each of their three worry topics for two minutes daily, and to record this on a homework diary sheet.

Participants attended again one week later for reassessment. Homework diaries were collected and participants repeated the PSWQ, STAI-T and LOT-R, followed by the breathing focus task. As an indirect check on adherence, participants' ability to reproduce the required daily practice was assessed by asking them to repeat the homework without reminders or prompting, after which they rated the verbal/imagery content and proportion of positive thoughts experienced. To examine participants’ ability to disengage from worry they rated how often (0 Never-10 Very often) they could stop worries that occurred outside of the homework exercise period. Participants were not explicitly asked to continue practicing the exercise after session 2. A month after this reassessment session participants completed (by mail) the PSWQ and STAI-T.

## 3 Results

### 3.1 Manipulation check

Session 1 involved training in the designated thinking style. The criterion for successful completion of the exercise was a score of 6 or higher (on a 10 point scale) in engaging with the designated thinking style (imagery or verbal) and 60% benign mentation (% positive and neutral content). During session 1 practices using the participant's worry topics, all groups were able to use their designated thinking style well above the cutoff (PIW: *M* = 8.10, *sd*. = 1.37; PVW: *M* = 8.45, *sd.* = 1.44; PIN: *M* = 7.83, *sd.* = 1.58). A one-way ANOVA revealed there were no significant differences between groups, *F* (2, 99) = 1.57, *ns*. All groups were also found to produce benign valence content above the cutoff point (PIW: *M* = 87.51, *sd.* = 9.72; PVW: *M* = 83.93, *sd.* = 12.00; PIN: *M* = 89.03, *sd.* = 9.11), with no significant differences between them, *F* (2, 99) = 2.23, *ns*.

A manipulation check was also administered during session 2 in the form of an adherence check, with mean scores on the above ratings again well above cut-offs, with no differences between groups. For designated thinking style (PIW: *M* = 8.83, *sd*. = 1.29; PVW: *M* = 8.93, *sd.* = 1.13; PIN: *M* = 8.36, *sd.* = 1.19), there were no significant differences between groups, *F* (2, 99) = 2.25, *ns*, nor for benign content (PIW: *M* = 94.78, *sd*. = 6.97; PVW: *M* = 94.57, *sd.* = 8.38; PIN: *M* = 94.03, *sd.* = 6.26), *F* (2, 99) = .10, *ns*.

### 3.2 Breathing focus task

The number of negative thought intrusions was analyzed in a mixed-model ANOVA, with one group factor (PIW, PVW, PIN) and two repeated factors: time (session 1–2) and assessor (self, independent). There was a significant main effect of time, λ = .52*, F* (1, 99) = 92.94, *p* < .001, η_p_^2^ = *.48*, with fewer negative intrusions reported in session 2 (3.6 down to 1.7). However, there were no significant main effects for group or rater, nor any significant interactions.

A Chi-square analysis similarly failed to reveal any differences between groups in the proportion of thoughts classified as low, medium or high in negativity by an independent rater (Preacher, 2001). For the post-intervention breathing focus task, medium and high negativity thoughts were collapsed due to low numbers of highly negative thoughts. The proportion of low versus medium/high negativity thoughts did not differ by group, χ^2^ (2, *N* = 206) = 2.73, *ns*. Across all groups, negative intrusions reduced over time (from 194 to 98 for low and 203 to 108 for medium/high negativity respectively), but the proportion of low to medium/high did not change, χ^2^ (1, *N* = 603) = .90, *ns*. In summary, all negative thoughts reduced over time, with no apparent differences due to group.

### 3.3 Effects of training on worry and anxiety

Mixed model ANOVAs were carried out on PSWQ and STAI-T scores, with one group factor and one repeated measures factor of time (session 1, session 2, follow-up). For the PSWQ, the only significant finding was a main effect of time, λ = .40, *F* (2, 98) = 74.40, *p* < .0005, η_p_^2^ = .50, with all groups showing reductions in worry across time (see Table 2 for means). Paired-samples t-tests revealed significant decreases from session 1 to 2, *t* (101) = 8.64, *p* < .001, and session 2 to follow-up, *t* (101) = 6.47, *p* < .001. Similarly, for the STAI-T, the only significant finding was a main effect of time, λ = .52, *F* (2, 98) = 45.61, *p* < .0005, η_p_^2^ = .38, with significant decreases from session 1 to 2, *t* (101) = 6.34, *p* < .001, and session 2 to follow-up, *t* (101) = 5.73, *p* < .001. Analysis of the LOT-R (a measure of optimism) assessed at session 1 and 2 only, also revealed a main effect of time, λ = .69, *F* (1, 99) = 43.64, *p* < .0005, η_p_^2^ = .29, with increased optimism overall, but no other significant effects. Thus, in all groups, worry and trait anxiety decreased significantly over time, while positive feelings of optimism increased, without any indication that this effect differed across conditions (all interaction *F*'s < 1).

The unexpected lack of group differences in worry or anxiety raises the question of whether, in the absence of a non-intervention control, all our conditions were equally effective, or equally ineffective. We therefore compared the effect size of changes observed in the present groups with those reported for non-treated control groups in two recent meta-analyses of psychological treatment for GAD (Cuijpers et al., 2014, Hanrahan et al., 2013). Studies were identified in which means and standard deviations were reported for non-treated groups over a follow-up period (ranging from 4 to 16 weeks) and in which participants completed the STAI-T (8) and/or the PSWQ (12). The average within-group effect size (Cohen's d) in these untreated groups was .10 for the STAI-T (ranging from −.27 to .32, *sd*. 0 .20) and .03 for the PSWQ (ranging from −.72 to .41, *sd*. .28). The effect size for the equivalent changes observed in the present study for the STAI-T ranged from .91 for the PIW group, to 1.01 for the PIN group, and 1.07 for the PVW group, giving an overall effect size of 1.0 for all participants; and for the PSWQ from 1.52 for the PIN group, to 1.86 for the PIW group, and 2.50 for the PVW group, giving an overall effect size of 1.92. The effects observed in the present study were thus well outside the range reported for untreated control groups, indicating that all the present interventions were indeed effective.

### 3.4 Post-hoc investigation of process variables associated with reduction in worry

Given there were no differences in outcome among the present groups, post-hoc analyses reported below combined all groups to identify relevant process variables.

#### 3.4.1 Negative thought intrusions during breathing focus

Since there was no significant effect of rater, the number of negative thought intrusions during session 2 was averaged across self and assessor. Mean negative intrusions were significantly correlated with PSWQ at follow-up, *r* = .27, *p* < .01. To explore this finding further, stepwise regression was conducted predicting PSWQ follow-up scores, entering baseline PSWQ and negative intrusions at step one, with session 2 negative intrusions added at step two. PSWQ and negative intrusions at baseline accounted for 15.8% of the variance and intrusions at session 2 predicted an additional 4.6% of the variance, *R* squared change = .046, *F* change (1, 98) = 5.70, *p* < .05.

Negative intrusions at session 2 were correlated with STAI-T trait scores at follow-up, *r* = .32, p < .05. As before, in regression analysis predicting final STAI-T, baseline anxiety and average negative intrusions accounted for 30.7% of the variance, but entering negative intrusions in step two did not significantly improve the prediction, *R* squared change = .018, *F* change (1, 98) = 2.59, *p* = .11. Hence, while fewer negative intrusions following homework practice predicted greater reductions in worry on the PSWQ, this effect did not generalize to trait anxiety.

#### 3.4.2 Ability to generate positive thoughts

The percentage of positive thoughts generated during the adherence check in session 2 was also correlated with PSWQ at follow-up, *r* = −.28, *p* < .005. In regression analysis predicting follow-up PSWQ, baseline PSWQ and the average percentage of positive thoughts generated in the practice scenarios in session 1 accounted for 16% of the variance. In step two, entering the percentage of positive thoughts at session 2 adherence check accounted for an additional 6.8% of the variance in final PSWQ, *R* squared change = .068, *F* change (1, 98) = 8.59, *p* < .01. Similarly, the correlation between positive thoughts generated in session 2 and STAI-T scores at follow-up was *r* = −.25, *p* < .05. Entering baseline STAI-T trait score and percentage of positive thoughts in the practice scenarios at step one explained 31.5% of the variance in final STAI-T, and adding positive thoughts from session 2 explained an additional 2.7%, *R* squared change = .027, *F* change (1, 98) = 3.99, *p* < .05.

#### 3.4.3 Ability to disengage from worry

During session 2 reassessment participants rated how often they had been successful in terminating any spontaneously occurring worries during the week. The correlation between this rating and PSWQ score at follow-up was *r* = −.23, *p* < .05. Hierarchical regression, after baseline PSWQ was entered in the first step as before, showed that entering disengagement from worry in step two accounted for a further 4.4%, *R* squared change = .044, *F* change (1, 99) = 5.42, *p* < .05. Similarly, after entering baseline STAI-T trait scores at step one, adding rated ability to shift away from worries in step two explained an additional 4.1%, *R* squared change = .041, *F* change (1, 99) = 6.26, *p* < .05. These results suggest that, in addition to involuntary thought intrusions during breathing focus, ability to voluntarily generate positive thoughts and disengage from worry predicted greater decreases in worry and anxiety, regardless of condition.

## 4 Discussion

We report here the first study of GAD assessing the effects of manipulating imagery and verbal processing in the longer term reduction of worry and anxiety. The main finding was that all three groups showed significant reductions in negative intrusions, and reported worry and anxiety, with no significant differences between conditions. Unexpectedly, the control condition in which participants practiced positive imagery chosen to be unrelated to worry content did not differ significantly from the conditions that involved practicing alternative positive outcomes of worry topics, whether in verbal or imagery form. Thus, it seems that the critical mechanism underlying the observed changes was replacing the usual flow of verbal worry with *any* alternative positive ideation. This suggests that, even if the negative and verbal form of worry contributes to its persistence, it is not necessary to directly modify this content to produce improvement.

Consistent with this interpretation, although negative intrusion frequency was substantially reduced, when intrusions did recur, they were still rated as moderately or highly negative. In other words, practicing any positive ideation reduced the *frequency* of worry-related thoughts, but not their negativity. Furthermore, reduced worry at follow-up was predicted by fewer negative intrusions during breathing focus, and greater ability to generate positive thoughts and disengage from worry in session 2. The lack of overall differences between groups, together with these *post-hoc* findings, again suggests that rather than reducing negativity of worry, the improvements were due to improved ability to disengage from it and focus instead on more positive content. These findings converge on the idea that repeated practice in replacing worry with positive ideation can counter the intrusive and distressing properties of worry.

One challenge to this conclusion is that, in the absence of a non-intervention control, the changes would have occurred without any intervention. We have argued that this is unlikely, given that the effect sizes were large and much greater than would be expected in the absence of any treatment. Even so, we cannot conclude from the present results that it is necessary to replace worry with *positive* ideation, because we did not include a non-positive condition. Even instructions to *imagine* negative outcomes, rather than the usual quasi-verbal form, reduces later intrusive thoughts (Hirsch et al., 2015, Stokes and Hirsch, 2010), perhaps because the more concrete content of images leads to outcomes being seen as more manageable or implausible. However, only conditions involving substituting *positive* content had the additional effect of reducing the perceived cost of worry outcomes, and enhancing perceived ability to cope (Hirsch et al., 2015).

Given that the present results were not compared with established treatments, we can make no claims for clinical effectiveness, nor would we suggest that the methods used here can be utilized as stand-alone interventions. The GAD volunteers in this study were not seeking treatment, so the utility of these methods in a clinical population is yet to be established. However, participants reported substantial improvements on measures of worry (e.g. within-group effect sizes of around 2 on the PSWQ), and these effects were maintained one month later. One clinical implication deserving further evaluation is that it may not be necessary to modify worry-related thought content directly, as is the aim of thought challenging in Cognitive Behavior Therapy. Future research could usefully compare the effectiveness of challenging negative thoughts versus practice in replacing them with any positive (or other) alternative. The latter approach may reduce negative intrusive thoughts and prevent consequent development of worry episodes, by increasing the availability of competing thoughts. At the very least, the present results indicate the need for research investigating whether modifying negative content, or enhancing access to positive alternatives, are equally or differentially effective in preventing uncontrollable worry in GAD.

## 5 Author note

This research was supported by grants from The Wellcome Trust (WT083204) and The Psychiatry Research Trust. The last author receives salary support from the National Institute for Health Research (NIHR), Mental Health Biomedical Research Centre at South London and Maudsley NHS Foundation Trust and King's College London. The views expressed are those of the authors and not necessarily those of The Wellcome Trust, The Psychiatry Research Trust, NHS, the NIHR or the Department of Health. Claire Eagleson is now at UNSW Australia. The authors would like to thank Ellena Cooke, Priya Kochuparampil, Zoe Maiden and Marc Williams for completing the assessor ratings and for Lewis Owens in help with referencing.

## Conflict of interest

The authors do not have any conflict of interests to declare.

## Figures and Tables

**Table 1:** Mean (SD) participant characteristics.

	Positive imagery	Positive verbal	Non-worry imagery
Age	28.84 (8.45)	31.20 (11.2)	30.83 (11.47)
Education (years)	15.56 (1.64)	15.21 (2.36)	14.91 (2.22)
PSWQ	69.44 (5.53)	68.69 (5.01)	68.77 (6.56)
STAI- T	59.78 (8.02)	60.29 (7.16)	61.54 (7.50)
LOT-R	8.78 (4.12)	8.80 (4.56)	7.89 (4.35)
BDI-II	24.31 (8.32)	26.51 (8.53)	29.30 (12.38)
Negative Intrusions (Breathing focus)	3.53 (2.12)	3.42 (2.29)	3.80 (2.73)

PSWQ= Penn State Worry Questionnaire; STAI-T = State Trait Anxiety Index- Trait version; LOT-R = Life Orientation Test- Revised version; BDI-II = Beck Depression Inventory-II.

**Table 2:** Mean (SD) scores on the PSWQ, STAI-T and LOT-R at each time point.

	Baseline	Session 2 (1 week post baseline)	Follow-up (5 weeks post baseline)
PSWQ			
Positive imagery	69.44 (5.53)	63.27 (6.79)	59.17 (8.90)
Positive verbal	68.69 (5.01)	60.06 (8.86)	56.14 (10.59)
Non-worry imagery	68.78 (6.56)	64.11 (7.78)	58.80 (9.13)
*All*	68.95 (5.70)	62.46 (8.00)	58.00 (9.59)
STAI-T			
Positive imagery	59.78 (8.02)	55.48 (8.49)	52.51 (8.84)
Positive verbal	60.29 (7.16)	56.49 (7.21)	52.60 (8.63)
Non-worry imagery	61.54 (7.50)	57.96 (9.07)	53.97 (9.23)
*All*	60.56 (7.52)	56.68 (8.27)	53.04 (8.84)
LOT-R			
Positive imagery	8.78 (4.12)	10.53 (4.14)	
Positive verbal	8.80 (4.56)	10.43 (4.37)	
Non-worry imagery	7.89 (4.35)	10.03 (4.50)	
*All*	8.48 (4.33)	10.32 (4.19)	

Note: PSWQ= Penn State Worry Questionnaire; STAI-T = State Trait Anxiety Index- Trait version; LOT-R = Life Orientation Test- Revised version. High scores on LOT-R indicate optimism.

## References

[bib1] Beck A.T., Steer R.A., Brown G.K. (1996). Manual for the Beck Depression Inventory-II.

[bib2] Borkovec T., Alcaine O., Behar E., Heimberg R.G., Turk C.L., Mennin D.S. (2004). Avoidance theory of worry and generalized anxiety disorder. Generalized anxiety disorder: advances in research and practice.

[bib3] Borkovec T., Inz J. (1990). The nature of worry in generalized anxiety disorder: a predominance of thought activity. Behaviour Research and Therapy.

[bib4] Butler G., Wells A., Dewick H. (1995). Differential effects of worry and imagery after exposure to a stressful stimulus: a pilot study. Behavioural and Cognitive Psychotherapy.

[bib5] Cuijpers P., Sijbrandij M., Koole S., Huibers M., Berking M., Andersson G. (2014). Psychological treatment of generalized anxiety disorder: a meta-analysis. Clinical Psychology Review.

[bib6] First M., Gibbon M., Spitzer R.L., Williams J. (1996). User's guide for the structured clinical interview for DSM-IV Axis I disorders—research version.

[bib7] Freeston M.H., Dugas M.J., Ladouceur R. (1996). Thoughts, images, worry, and anxiety. Cognitive Therapy and Research.

[bib8] Hanrahan F., Field A.P., Jones F.W., Davey G.C. (2013). A meta-analysis of cognitive therapy for worry in generalized anxiety disorder. Clinical Psychology Review.

[bib9] Hayes S., Hirsch C.R., Mathews A. (2010). Facilitating a benign attentional bias reduces negative thought intrusions. Journal of Abnormal Psychology.

[bib10] Hirsch C.R., Hayes S., Mathews A., Perman G., Borkovec T. (2012). The extent and nature of imagery during worry and positive thinking in generalized anxiety disorder. Journal of Abnormal Psychology.

[bib11] Hirsch C.R., Mathews A. (2012). A cognitive model of pathological worry. Behaviour Research and Therapy.

[bib12] Hirsch C.R., Mathews A., Lequertier B., Perman G., Hayes S. (2013). Characteristics of worry in generalized anxiety disorder. Journal of Behavior Therapy and Experimental Psychiatry.

[bib13] Hirsch C.R., Perman G., Hayes S., Eagleson C., Mathews A. (2015). Delineating the role of negative verbal thinking in promoting worry, perceived threat and anxiety. Clinical Psychological Science.

[bib14] Leigh E., Hirsch C.R. (2011). Worry in imagery and verbal form: effect on residual working memory capacity. Behaviour Research and Therapy.

[bib15] Meyer T.J., Miller M.L., Metzger R.L., Borkovec T.D. (1990). Development and validation of the Penn State Worry Questionnaire. Behaviour Research and Therapy.

[bib16] Philippot P., Baeyens C., Douilliez C. (2006). Specifying emotional information: regulation of emotional intensity via executive processes. Emotion.

[bib17] Preacher K.J. (2001). Calculation for the chi-square test: An interactive calculation tool for chi-square tests of goodness of fit and independence [computer software]. http://quantpsy.Org.

[bib18] Scheier M.F., Carver C.S., Bridges M.W. (1994). Distinguishing optimism from neuroticism (and trait anxiety, self-mastery, and self-esteem): a reevaluation of the Life Orientation Test. Journal of Personality and Social Psychology.

[bib19] Spielberger C., Gorsuch R., Lushene R., Vagg P., Jacobs G. (1983). Manual for the State-Trait Anxiety Inventory.

[bib20] Stefanopoulou E., Hirsch C.R., Hayes S., Adlam A., Coker S. (2014). Are attentional control resources reduced by worry in generalized anxiety disorder?. Journal of Abnormal Psychology.

[bib21] Stöber J., Tepperwien S., Staak M. (2000). Worrying leads to reduced concreteness of problem elaborations: Evidence for the avoidance theory of worry. Anxiety, Stress & Coping: An International Journal.

[bib22] Stokes C., Hirsch C.R. (2010). Engaging in imagery versus verbal processing of worry: impact on negative intrusions in high worriers. Behaviour Research and Therapy.

[bib23] Williams M.O., Mathews A., Hirsch C.R. (2014). Verbal worry facilitates attention to threat in high-worriers. Journal of Behavior Therapy and Experimental Psychiatry.

